# A Novel Energy-Efficient Pyrolysis Process: Self-pyrolysis of Oil Shale Triggered by Topochemical Heat in a Horizontal Fixed Bed

**DOI:** 10.1038/srep08290

**Published:** 2015-02-06

**Authors:** You-Hong Sun, Feng-Tian Bai, Xiao-Shu Lü, Qiang Li, Yu-Min Liu, Ming-Yi Guo, Wei Guo, Bao-Chang Liu

**Affiliations:** 1 College of Construction Engineering, Jilin University, Changchun 130021, PR China; 2 Department of Civil and Structural Engineering, School of Engineering, Aalto University, PO Box 12100, FIN-02015 Espoo, Finland

## Abstract

This paper proposes a novel energy-efficient oil shale pyrolysis process triggered by a topochemical reaction that can be applied in horizontal oil shale formations. The process starts by feeding preheated air to oil shale to initiate a topochemical reaction and the onset of self-pyrolysis. As the temperature in the virgin oil shale increases (to 250–300°C), the hot air can be replaced by ambient-temperature air, allowing heat to be released by internal topochemical reactions to complete the pyrolysis. The propagation of fronts formed in this process, the temperature evolution, and the reaction mechanism of oil shale pyrolysis in porous media are discussed and compared with those in a traditional oxygen-free process. The results show that the self-pyrolysis of oil shale can be achieved with the proposed method without any need for external heat. The results also verify that fractured oil shale may be more suitable for underground retorting. Moreover, the gas and liquid products from this method were characterised, and a highly instrumented experimental device designed specifically for this process is described. This study can serve as a reference for new ideas on oil shale in situ pyrolysis processes.

The tremendous growth in global energy demand and the rapid depletion of conventional oil resources have created a demand for alternative energy sources. Oil shale^1^,^2^,^3^,^4^, an organic-rich petroleum source rock, has a high content of solid insoluble kerogen that can release a petroleum-like liquid, that is, shale oil, and provide secure access to transportation fuels^5^,^6^. The huge reserves of oil shale, approximately 35% of the world's total energy reserves^7^, make it commercially viable. Accordingly, scientists have been attempting for decades to develop environmentally responsible methods of exploiting oil shale^8^,^9^,^10^,^11^,^12^,^13^,^14^.

Oil shale can be used in various ways^15^,^16^,^17^,^18^,^19^,^20^, from electrical energy via direct combustion to a wide range of petrochemical products (including shale oil and other liquid fuels) via the pyrolysis of kerogen. Two primary types of processes for oil shale retorting have been developed: ex situ and in situ processes. Ex situ (aboveground) processes^16^,^20^, such as retorts of the Kiviter, Petrosix, ATP, Tosco-II and Fushun-type, are the primary extraction methods. However, such methods suffer critical problems^8^,^16^,^17^,^21^,^22^, including potentially harmful semicoke waste, large land use, and gas pollution. Additionally, ex situ processes are unsuitable for low-grade oil shale buried in deep formations because of mining difficulties and negative economic impacts. In situ processes^10^,^11^, such as ICP, Electrofrac™, Chevron's technology, and the Occidental Modified in-situ (MIS) process, have recently attracted great attention, without regard to the heating modes and environmental issues related to these methods. The ICP and Electrofrac™ processes use electricity to heat the surrounding shale deposit as a physical heat treatment, consuming significant energy^10^. Chevron's technology uses heated and pressurised carbon dioxide to heat the oil shale, which also requires large quantities of water and damages the environment. The MIS method induces combustion air to permeate and burn oil shale underground after mining 20% of the oil shale and fracturing the rest to create a void space of 20–25%; this method was conducted in situ in 1972 at Logan Wash, Colorado^10^.

We recently introduced the oil shale topochemical reaction^23^ as a low-energy shale oil recovery method that is triggered by the topochemical reaction between oil shale and a limited amount of air to drive the spontaneous pyrolysis of oil shale in the absence of an external heat supply (denoted as a self-pyrolysis process). In this method, the pyrolysis represents a chemical-enhanced heating process rather than a physical heating process, like ICP and Electrofrac™, or a complete combustion process. Moreover, the shale oil product still consists primarily of hydrocarbons, like the oils obtained from traditional processes. The proposed technique^23^ has the potential to consume much less energy, making it more suitable for underground retorting. However, neither the heat transfer mechanisms of the proposed method nor the importance of process design in realising energy savings has been elucidated. Furthermore, previous experiments have been limited to data obtained from a tube furnace for the analysis of products, with little data on heat transport properties. Results cannot therefore be directly applied to energy-efficient pyrolysis solutions. The purpose of this paper is to address these gaps. We aim to provide new insights into the mechanism of the topochemical-induced thermal process of oil shale conversion to gain a novel understanding of the self-pyrolysis process of oil shale and demonstrate a more feasible energy-efficient approach through a laboratory-scale process.

Oil shale generally exhibits poor thermal conductivity and permeability and is therefore very inefficient at transmitting heat and gas. For ex situ retorting, shale is always crushed into smaller particles (commonly < 100 mm) to increase its specific surface and contact areas. For in situ methods, porosity and permeability are two important factors that enable the transfer of heat to the shale within the geologic formation^14^. Many methods^14^,^24^,^25^, including the use of explosives, hydraulic fracturing, and horizontal drilling, can be used to rubblize the formation to improve the stratum permeability and increase the reaction area. In these experiments, various sizes (2–100 mm) of oil shale were introduced into a fixed bed to form a heterogeneous system to mimic different permeabilities and porosities of shale in the field.

In the present study, a self-pyrolysis retorting process of oil shale with a low energy input using high- and normal-temperature air in sequence as carrier gases is described. A highly instrumented fixed bed (Figure 1a) simulating the horizontal stratum was developed to investigate this novel pyrolysis in a porous medium under controlled conditions. The propagation of fronts formed in this process, the temperature evolution, and the reaction mechanism of oil shale pyrolysis in porous media are discussed and compared with those in a traditional oxygen-free process. Two different circular gas pipelines (Figures 1b, c) were specifically designed to simulate the in situ hydraulic fracturing and horizontal drilling modes. The products from this method were also characterised. We anticipate that this study can serve as a starting point to initiate new approaches to oil shale in situ pyrolysis processes.

## Results

### Thermogravimetric (TG) analysis, differential thermogravimetric (DTG) analysis and differential scanning calorimetry (DSC)

The results of the TG, DTG, and DSC experiments on Huadian (HD) oil shale under both air and inert (N_2_) atmospheres are shown in Figure 2. The experiments were repeated several times and exhibited good reproducibility. The stages are as follows.

- At temperatures less than 200°C, a small mass loss is observed, due primarily to the evaporation of water, including adsorbed and interlayer water, from clay minerals.
- Under an air atmosphere, a slight rebound is observed at approximately 300°C because of the absorption of ambient gas^17^; this result differs from the results obtained under an N_2_ atmosphere.
- In the temperature range from 300°C to 550°C, a major mass loss is observed. This stage is attributed primarily to the decomposition of kerogen into volatiles. Under an air atmosphere, the reaction is more rapid, and two peaks are observed in both the DTG and DSC curves. The first peak, referred to as the low-temperature oxidation peak, is observed at 336°C and is due to the oxidation reaction of light hydrocarbons formed by cracking. The second peak, referred to as the high-temperature oxidation peak, appears at 405°C and originates from the oxidation of heavy hydrocarbons, fixed carbon and possibly other components. The two processes together gave off 10.693 MJ kg^−1^ of heat, which is consistent with the proximate analysis results. However, these two exothermic peaks did not appear when the experiment was performed under N_2_, and the total endothermic heat was 1.191 MJ kg^−1^ under an N_2_ atmosphere.These results reveal that the exothermic reaction heat from oil shale oxidation is approximately nine times the heat required for oil shale pyrolysis. Therefore, the introduction of a reasonable amount of oxygen to partially oxidise the oil shale and release heat for the surrounding oil shale pyrolysis would facilitate the production of petrochemical goods from oil shale.
- At temperatures greater than 600°C, a final mass loss is observed because of the thermal decomposition of carbonates and clay minerals; the endothermic peak appears in two experiments. The endothermic quantity under air was 49.4% of that under N_2_.

### Evolution of temperature in oil shale self-pyrolysis

**Figure 1:**
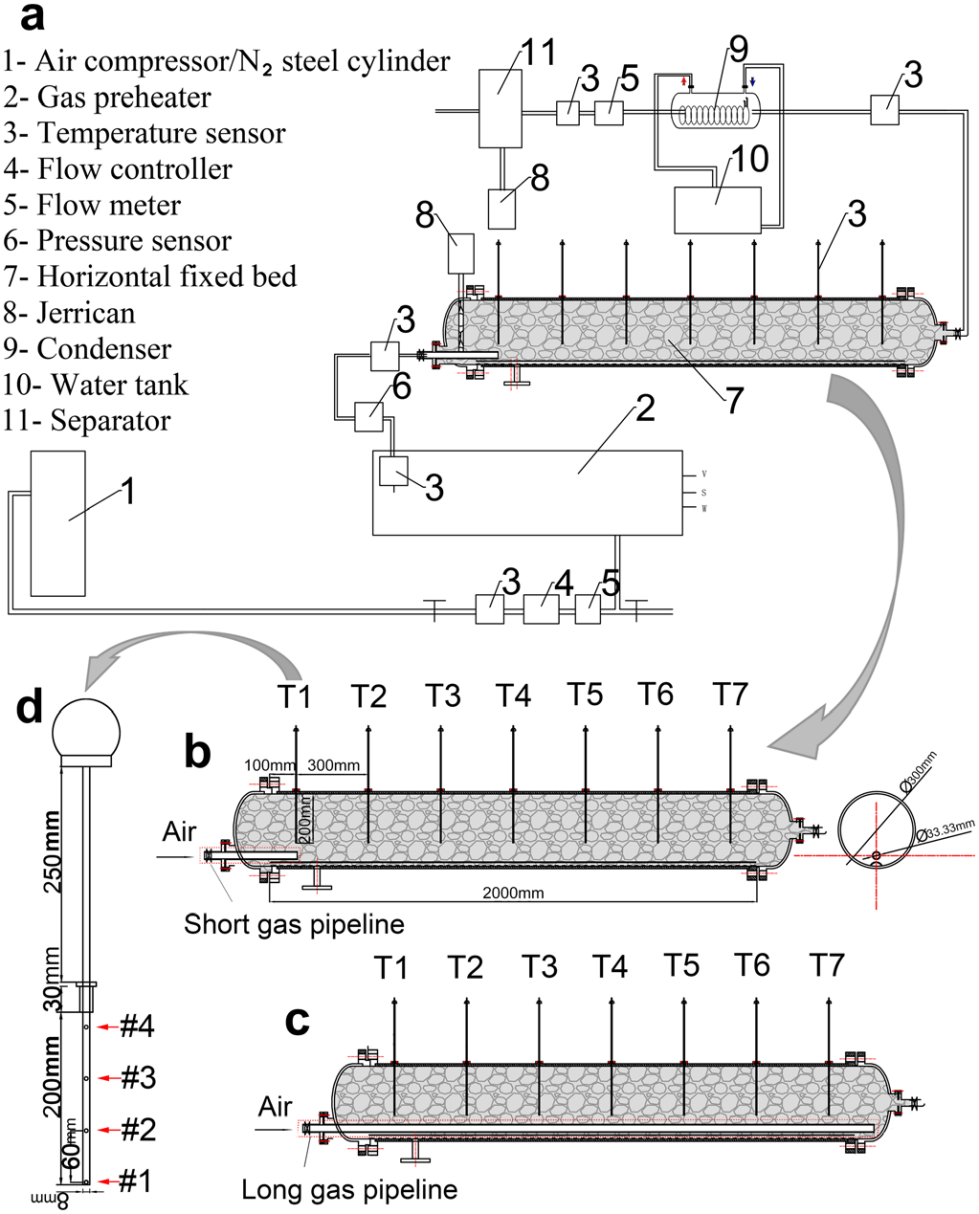
Schematic of the experimental device. (a), The overall layout of the device. (b), The fixed bed with short gas pipeline. (c), The fixed bed with long gas pipeline. (d), The temperature sensor.

The gas pipeline shown in Figure 1b was used for the oil shale self-pyrolysis experiments, and the rubblized oil shale was introduced into the bed to study its heat transfer process. An air compressor was used in these experiments to provide air. Air was preheated and maintained at 400–500°C with a flow rate of 16 m^3^ h^−1^ before being fed into the fixed bed. The temperatures at different locations inside the bed are reported in Figure 3a. For sensor T1, which was positioned close to the gas entrance, the temperature of T1-#1 (sensor location 1; sensor number 1) slowly increased to 220°C in the first 27 min before rapidly increasing to 600°C within 3 min, triggering a topochemical reaction in the oil shale. The temperature successively increased along the vertical direction of the gas pipeline, indicating that the front propagated as a vertical surface. The temperature propagation was consistent with the gas flow direction, i.e., co-current transmit (the reaction front and feeding gas take place on the same side of the bed), spreading from left to right along the horizontal direction of the gas pipeline.

Figure 3b clearly shows that the temperature is affected by the fluctuation in the gas flow and that it is characterised by a positive relationship. Temperatures increased as the gas flow increased and rapidly decreased as the flow decreased in all stages of the reaction. After the topochemical reaction is triggered, the temperature can reach 800°C or higher if the flow is excessive. As shown in Figure 3b, the temperature of T1-#1 quickly increased to 800°C in 33 min. This continuous high-temperature condition can consume a large amount of organic matter and is not beneficial to the extraction of shale oil. Conversely, the reactions will stop and the oil shale will cool if the gas source is cut off. A reasonable flow can maintain a steadily propagating front with a slowly increasing temperature, and the oil shale can be pyrolysed smoothly without external heat provision. This lack of need of external heat is due to the topochemical reaction between the oil shale and the oxygen from the air, which spontaneously increases the temperature of the oil shale in the beginning before driving the continuous pyrolysis of kerogen and accomplishing the retorting of the oil shale.

### Time-interval trials of oil shale self-pyrolysis

As shown in Figure 3c, three time-interval trials of self-pyrolysis were run to verify the feasibility and explore the mechanism of the proposed method. Hot air was firstly fed into the fixed bed. When the temperature of T1 increased to 250–300°C, the gas preheating was stopped and the hot gas was replaced by ambient-temperature air. The gas flow was adjusted simultaneously. After a given period of time, the gas source was shut off and the fixed bed was allowed to cool naturally. This trial process was repeated twice. The interval of the first discontinuation was 630 min, and the second (2520 min) was longer than the first to allow the fixed bed to completely cool.

As shown in Figure 3c, the pyrolysis was activated by hot air sweeping for 30 min in the first trials, whereas, in the second and third trials, much longer times were required to induce the reaction. The reason for the longer times was that, during the first trial, the oil shale near sensor T1 was pyrolysed and the front propagated and reached T2 (see Figure 3d), which led to the reduction of the thermal parameters of the residues (Table 1); this reduction resulted in more exchange heat being consumed by the residues during the initial stage of the second and third trials. However, the self-pyrolysis reaction of oil shale is easily triggered, and once started, it can spontaneously and smoothly propagate in the absence of any external heat supply.

The peak temperature of the #1 thermocouples reached in sequence, as shown in Figure 3d, again reveals that, for the bed equipped with a short pipeline, the pyrolysis fronts caused by the topochemical reaction were co-current with the feeding of air. The temperature fluctuation of the sensors shown in Figure 3d might be explained by the complex flow developed in the bed, which was caused by the conversion of solid kerogen into liquid oil and then into gas^26^,^27^, generating overpressure in the bed. The four thermocouples of all sensors reached their peak temperatures at approximately the same time, indicating that sharp fronts were generated during propagation. For all sensors except T1, the #3 thermocouple was always the first one to register a temperature increase and the #1 thermocouple always registered the highest temperature. Such a phenomenon could be caused by the flow's upward mobility and the significant heat loss at the wall of the bed.

### Evolution of temperature in an oxygen-free pyrolysis process

In a traditional oxygen-free oil shale pyrolysis experiment, the gas was replaced by hot nitrogen at two different speeds of 16 and 25 m^3^ h^−1^. Figure 3e shows the results for a gas flow of 25 m^3^ h^−1^. During this experiment, ten cylinders (4 m^3^ cylinder^−1^) of nitrogen were used; the small zigzags in the curves are associated with the replacement of the nitrogen cylinders. The nitrogen gas had been preheated before being fed into the fixed bed within 105 min; however, the highest temperature in this bed was only 325°C. When the nitrogen gas was no longer preheated, the fixed-bed temperature began to plunge, indicating that nitrogen was just a heat carrier. Moreover, the fixed bed could only reach a temperature of 76°C in 30 min when the gas flow rate was adjusted to 16 m^3^ h^−1^, as shown in Figure 3f.

### The structure of the reaction fronts for different gas pipelines

Horizontal drilling connecting two boreholes is regarded as a suitable method for oil shale in situ retorting, especially for a thinner shale layer, because it can increase the interaction between the gas and the oil shale layer, which is also beneficial to the transmission of the shale oil. In the present study, the gas channel shown in Figure 1c was designed to mimic the in situ horizontal drilling mode. With a long gas pipeline, the first sensor to register an increase was T7-#1 (located at the outlet of the bed), followed successively by T6-#1, T7-#2, T6-#2, T7-#3, and T6-#3. The highest entrance temperature near T1 was less than 150°C. The front propagation in the long pipeline, from right to left, was completely opposite to that of the short pipeline because it followed a counter-current mode (with the reaction front and feed of gas occurring on opposite sides of the bed). Moreover, the temperature of T4 increased slowly, and that of T3 required a longer time to increase.

### Analysis of the produced gas and liquid oil and the solid residue

In this part, the products of the oil shale self-pyrolysis were analysed. During each experiment, a total of 110–130 kg of oil shale was fed into the bed. The shale oil, water and residue collected from three separate and repeated experiments are reported in Figure 4a. Compared with the Fischer analysis of both virgin and spent oil shale results in Table 1, only a small amount of shale oil (an average of 1.9%) was consumed in the oil shale self-pyrolysis process with air. The amounts of water and gas increased and the amount of residue decreased because of the oxidation of fixed carbon and volatile substances. The main components of the products were analysed as follows.

A large quantity of pyrolysed gas was generated with the increase in temperature. The colour of the vapour changed to dense-white as the temperature increased, and a large amount of shale gas discharged even when the air was no longer supplied to the fixed bed. The gas exhibited a pungent odour and was flammable (Figure 4b). The shale gases were determined by gas chromatography (GC) (Figure 4d). The chromatograms revealed that the major hydrocarbon components were methane, ethylene, ethane, propylene, propane, butene, and butane.

Oil was gradually expelled from the bed into a jerrican during the experiment. When the experiment was stopped midway through, no liquid oil was observed in the porous medium. Like petroleum, shale oil (Figure 4c) is a black-brown material with an irritating odour. In addition to its use as a transport fuel^5^,^6^, shale oil can also serve as a virgin material for the production of numerous different value-added products^28^,^29^, such as rubber softeners, chemical intermediates, and asphalt additives.

TG analysis under N_2_ (Figure 4e) was carried out primarily to study the characteristics of the boiling point/range of the oil samples. The mass loss between the ambient temperature and 400°C was primarily due to the distillation of the volatile hydrocarbons at 20–280°C and of the low-molecular-weight hydrocarbons at 280–400°C; the mass loss between 400 and 500°C was due to a combination of distillation and thermal cracking of medium-molecular-weight hydrocarbons^30^,^31^,^32^. In this study, the mass loss at 20–280°C was 85%, indicating that the shale oil obtained from our experiments was primarily volatile hydrocarbons. A small amount of residue, approximately 7%, remained in the pan even after the sample was to above 600°C, similar to the residue observed for crude oil^30^. The GC results for the liquids (Figure 4f) show that numerous hydrocarbons and derivatives were present in the collected oils, mainly as aliphatic (63.66%), aromatic (22.80%), and heteroatom (13.54%) compounds. Among these hydrocarbons, aromatic hydrocarbons were primarily produced in the first 16 min and aliphatic hydrocarbons were dominated by a bimodal distribution of *n*-alkane and *n*-alkene doublets that extended up to C_34_.

## Discussion

A low-energy-cost strategy for oil shale pyrolysis has been developed to optimise oil shale retorting technology. Different from the typical methods that use hot nitrogen as the carrier gas, the new approach is based on chemical-enhanced heating derived from the topochemical reaction inside the oil shale under preheated air. Thermal analysis shows that, after the topochemical reaction has been triggered by preheated air, it can provide sufficient heat for the subsequent pyrolysis without external heat consumption. Thus, it is a highly energy-efficient self-pyrolysis process compared to the conventional methods. Moreover, an experimental device was designed and constructed to perform this low-energy-cost oil shale pyrolysis in the lab.

The aforementioned results provide new insights into the mechanisms of topochemically induced heating for oil shale pyrolysis, which are critically important for determining the energy required for completing the pyrolysis without external heat. As shown in Figure 5, multiple zones were identified in the bed: the spent shale zone, the retorting zone, and the preheating zone. In the spent shale zone, the heat generated from topochemical reactions between oxygen, water and fixed carbon or that generated from produced gas^23^ is applied to the subsequent oil shale pyrolysis in the retorting zone. In the retorting zone, the organics or kerogen is pyrolysed into hydrocarbons, producing fixed carbon. In the preheating zone, the oil shale is preheated by the residual heat from the exhaust gases. However, the reactions in the bed are complex, as each zone gradually undergoes a transition along the fixed bed, and the self-pyrolysis propagation is the result of a coupling between oil shale pyrolysis, oxidation and the transfer of heat and gas. Heat conduction and convection are the main modes of heat transfer^33^.

**Figure 2:**
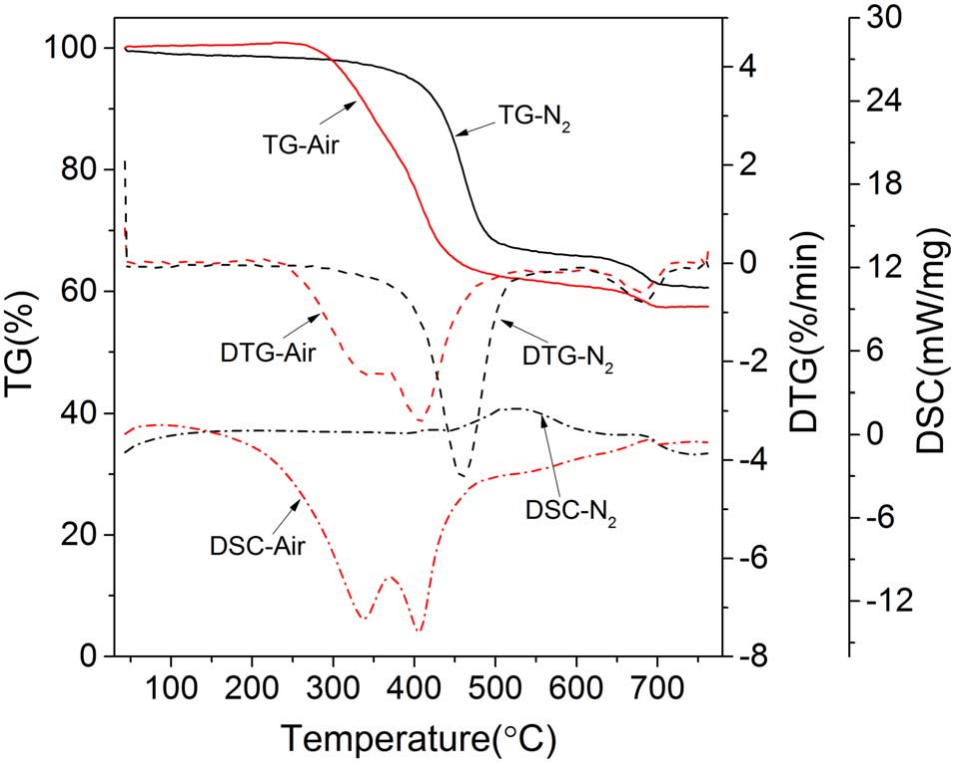
TG/DTG/DSC results for virgin HD oil shale under air and under nitrogen.

**Figure 3:**
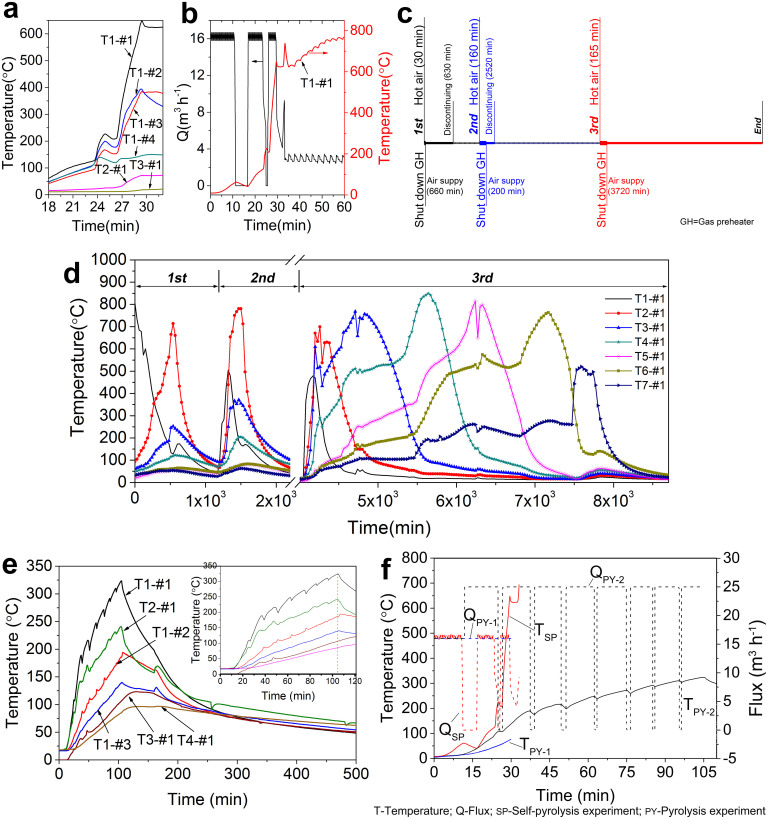
The temperature evolution curves and experimental timeline. (a), Temperature evolution of the thermocouples in the self-pyrolysis experiment between 18 and 33 min. (b), Interaction between temperature evolution and gas flow. (c), Timeline of time-interval trials. (d), Temperature evolution of the #1 thermocouples during the time-interval of the self-pyrolysis trials. (e), Temperature evolution of the thermocouples in the pyrolysis process (25 m^3^ h^−1^). (f), Temperature evolution of thermocouple T1-#1 in different experimental processes.

**Figure 4:**
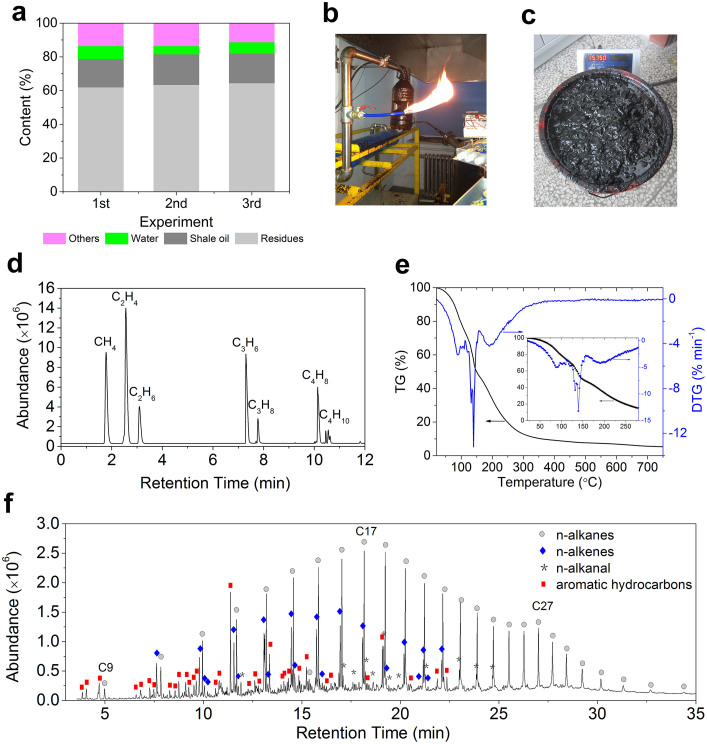
The product analysis. (a), The yield of products from the self-pyrolysis experiments. (b), Shale gas ignition tests. (c), Shale oil collected during the experiments. (d), Chromatogram of gas collected from an experiment. (e), TG/DTG analysis of the shale oil. (f), GC-MS spectrum of shale oil collected from the experiment.

**Figure 5:**
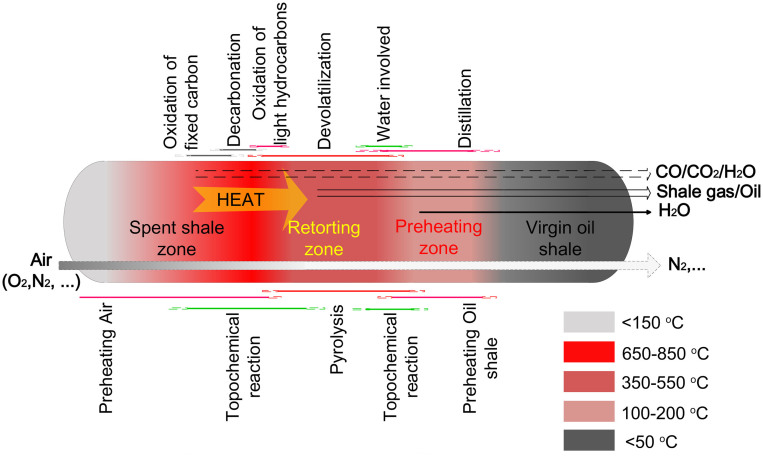
Description of the main phenomena occurring during the propagation of the self-pyrolysis front.

The topochemical reaction runs through the entire process, especially in the preheating and spent shale zones. In the preheating zone (Figure 5), oil shale is preheated to less than 200°C, producing water and carbon dioxide^23^. The water-filled pores in oil shale then form an autocatalytic system at 200–300°C, reducing the energy needed for chemical bond breakage and accelerating the conversion of kerogen into bitumen and into oil/gas. In the spent shale zone, with adequate oxygen, abundant heat is released through the complete oxidation of light hydrocarbons and the temperature increases rapidly (Figures 3a, b), accompanied by incomplete oxidation in areas with low concentrations of oxygen. Further oxidation of the fixed carbon consumes a large amount of oxygen, resulting in the temperature increasing to 800°C or higher^3^. This series of reactions may play a key role in the self-pyrolysis reaction process, which can be confirmed in time-interval trials. In the third trial, the temperatures of T1 and T2 increased to 500–700°C even after the oil shale completely pyrolysed in the previous two trials. Moreover, as evident in Figure 3d, when the sensor reached the highest temperature (~800°C), its right-side sensor was approximately 500°C; thus, according to the TG/DTG analysis in Figure 2, pyrolysis of the oil shale near the right-side sensor occurred. The small amount of fixed carbon (0.22%) in the residue also verified that almost all of the fixed carbon was consumed. All of these exothermic oxidations provided sufficient energy for further reactions and zone expansion. Moreover, the decomposition of kerogen, accompanied by changes in the microstructure of the solid phase, increased the porosity and opened channels for oil/gas drainage, which could improve gas transfer and heat conduction. In our contrast experiment, under an N_2_ atmosphere, the supply of external heat was required through the whole pyrolysis of oil shale (~500°C); otherwise, the pyrolysis was terminated in a short time. The potential heat of the fixed carbon contained in the shale coke is not used, influencing the thermal efficiency of the process^34^.

The topochemical reactions of oil shale can be easily triggered and repeated. After they have been triggered, oil shale can undergo pyrolysis spontaneously and smoothly with a limited amount of oxygen in the absence of external heat provision and the propagation speed of the pyrolysis front can be controlled by the air supply velocity. Compared with conventional oxygen-free pyrolysis, the heat produced by the topochemical reactions among the oxygen, water, fixed carbon and organics retained in the oil shale can not only accelerate the decomposition of kerogen but can also greatly reduce production costs. Moreover, the pyrolysis of oil shale triggered by topochemical heat is superior in its rapid heating rate and lower preheating gas requirement with a lower flow rate (Figure 3f). Hence, the low energy input and simplified process, together with the high extraction yield of the shale oil/gas, indicate that the proposed method is economically favourable towards retorting and in situ conversion.

Comparing the propagating fronts of the two experimental gas pipelines, we observed that, for the long gas pipeline, the hot gas flowed over the entire pipe until the end of the bed, at which point convective heat transfer with the surrounding shale occurred. As the temperature of T7 increased, the topochemical reaction started and heat was produced; the reaction fronts and gas flow acted in opposite directions. However, for approximately half of the experiment, the block of temperatures spread around T4 indicated that the gas was not only a reactant but also a heat carrier. The counter-current flow mode in the bed appears not to enhance the gas flow and the heat transfer; thus, the topochemical reaction gradually ceases. Therefore, horizontal drilling of a completely open channel may not necessarily be suitable for oil shale underground retorting. Hydraulic fracturing, which creates high-conductivity fissures with large areas in the oil shale stratum, appears to be more conducive to gas transmission and heat exchange.

The low-energy-consumption method described in this study provides an alternative route for further in situ industrial applications because of the possibilities of significant scale-up and lower environmental pollution. Notably, however, numerous critical issues must be considered before self-pyrolysis technology can be used for in situ industrial production. Firstly, further research should include a general performance evaluation and a comparative assessment of its energy use and cost. To ensure that the proposed self-pyrolysis is designed to maximise the topochemical reaction heat, a more detailed thermal analysis, including peak temperature and all of the incremental changes in the process parameters in all stages, should be conducted. For example, the peak temperature in our experiment was 800°C or higher (Figures 3b, d); however, the process could be optimised by limiting the temperatures to 600°C. The issue of how to provide sufficient heat through a complex in situ fracture network to trigger the reaction and drive the self-pyrolysis process also requires investigation. Moreover, the optimisation of oxygen content and gas flow for oil shale self-pyrolysis to improve the energy efficiency and reduce the costs is also necessary; such work is currently in progress.

## Methods

### Materials

The oil shale used was obtained from the Gonglangtou mine located in Huadian (HD), China. The physical properties of the virgin oil shale and the spent oil shale from the self-pyrolysis experiments are summarised in Table 1.

### Experimental apparatus

The experimental system shown in Figure 1a was specifically designed to enable the proposed self-pyrolysis experiments to be carried out. This system consisted of four main components: a gas supply and heating device, a fixed bed, a separation and recovery device, and a data monitoring and collection device. The gas supply and heating device comprised an air compressor or N_2_ steel cylinder and a gas preheater. The fixed bed consisted of a horizontal cylindrical reactor chamber with an internal diameter of 325 mm and length of 2 m. The diameter was chosen to be sufficiently wide to limit heat losses from the walls and to facilitate treatment of the fuel gas. In addition to the gas channel, a groove was cut into the bottom of the bed to allow shale oil to discharge into a jerrican. The whole fixed bed can pack approximately 0.15 m^3^ of oil shale. The fixed bed was constructed of a 5 mm thick stainless–steel material without a heating system and was surrounded by a 50 mm thick layer of refractory fibre. The separation and recovery device comprised a condenser, an oil and water separator, a jerrican and a water tank. The data monitoring and collection device comprised a flow meter, a temperature sensor, a pressure sensor and a paperless recorder.

### Gas channel

Embedded gas channels were equipped into the fixed bed; these channels allowed the heated gas to enter into the bed. In this experiment, two circular gas pipelines with different lengths were used, as shown in Figures 1b, c. The short gas pipeline (Figure 1b) only reached the first sensor, whereas the long gas pipeline (Figure 1c) passed through the whole fixed bed, simulating the horizontal drilling mode. The external diameter of these two pipelines was 70 mm. The surface of both pipelines was full of long and narrow slits, which were beneficial to uniform gas discharge and could also prevent oil shale particles from falling into the pipeline. The short gas pipeline was generally used and is referred to as the default pipeline.

### Temperature measurements

The fixed bed was finely instrumented, as shown in Figure 1. A group of seven in-line temperature sensors with a diameter of 8 mm and a length of 480 mm was equally spaced in the horizontal direction (from the left to the right of the reactor: T1, T2, T3, T4, T5, T6 and T7, as shown in Figures 1b, c), extending 200 mm into the reactor. At the length of 200 mm, four thermocouples (Figure 1d) were located at z = 0, 60, 120 and 180 mm (from the bottom to top of the sensor), enabling measurements of the temperature along the radial and axial cross-sections of the whole bed and monitoring of the change in temperatures and the progress of the front. Before the experiment, the temperature sensors were calibrated using a tubular quartz reactor; the range of temperature error was 2%, satisfying the precision requirements of our test.

### Self-pyrolysis experiments

Firstly, a mass of large rubblized oil shale (2–100 mm, 110–120 kg), mimicking an anisotropic artificial fracture, was introduced into the fixed bed. For the self-pyrolysis experiments, hot air preheated by the gas preheater from ambient temperature to 400–500°C was fed into the bed via the gas pipeline. After the topochemical reaction was triggered, the heater was closed and the hot gas was replaced by ambient-temperature air. For the traditional oxygen-free pyrolysis process, pure hot N_2_ was used as the only carrier gas. During the experiments, the gas flow was adjusted to control the reaction.

### Analytical methods

TG and DSC were performed using a Netzsch STA449F3 (Germany) with a heating rate of 10°C min^−1^ under both N_2_ and air atmospheres (50 mL min^−1^). The GC-MS analyses were carried out using an Agilent 6890/5973 N GC-MS instrument (America).

## Author Contributions

Y.H.S., F.T.B. and Q.L. designed the research; Y.H.S., F.T.B., Q.L. and B.C.L. designed the experimental device; F.T.B., Q.L. and Y.M.L. performed the experiments; Y.H.S., F.T.B., X.S.L., Q.L., Y.M.L., M.Y.G., W.G. and B.C.L. analysed the data. Y.H.S., F.T.B. and X.S.L. wrote this manuscript; all authors read and edited the manuscript.

## Figures and Tables

**Table 1:** Physical properties of the HD oil shale

	Virgin oil shale	Spent shale
*Density (kg m^−3^)*	1537	790
*Proximate analysis (wt.%, ad ^a^)*		
Volatiles	34.46	1.76
Fixed carbon	3.75	0.22
Ash	61.80	98.46
Moisture (as received)	5.53	0.59
Calorific value (MJ kg^−1^)	11.21	0.42
*Ultimate analysis (wt.%, ad)*		
C	26.38	0.79
H	4.16	0.17
N	0.31	0
S	1.04	2.21
*Fischer assay analysis (wt.%, ad)*		
Shale oil	19.69	0.87
Gas	6.77	1.15
Water	6.92	1.8
Residue	66.62	96.18
*Thermal properties*		
Thermal conductivity (W m^−1^K^−1^)	0.68	0.17
Thermal diffusivity (mm^2^ s^−1^)	0.50	0.68
Specific heat (MJ m^−3^K^−1^)	1.36	0.26
*Porosity (%,ad)*	1.44	58.98
*Permeability (mD, ad)*	0.002	0.395

ad: air dry basis.
